# Defensive coping and health-related quality of life in chronic kidney disease: a cross-sectional study

**DOI:** 10.1186/1471-2369-12-28

**Published:** 2011-06-20

**Authors:** Anna Kaltsouda, Petros Skapinakis, Dimitrios Damigos, Margarita Ikonomou, Rigas Kalaitzidis, Venetsanos Mavreas, Kostas C Siamopoulos

**Affiliations:** 1 Department of Psychiatry, School of Medicine, University of Ioannina, Ioannina 45110, Greece; 2 Psychonephrology Unit, Department of Nephrology, School of Medicine, University of Ioannina, Ioannina 45110, Greece; 3 Department of Nephrology, School of Medicine, University of Ioannina, Ioannina 45110, Greece

## Abstract

**Background:**

Coping with the stresses of chronic disease is considered as a key factor in the perceived impairment of health related quality of life (HRQL). Little is known though about these associations in chronic kidney disease (CKD). The present study aimed to investigate the relationship of defensive coping and HRQL among patients in different CKD stages, after adjusting for psychological distress, sociodemographic and disease-related variables.

**Methods:**

The sample consisted of 98 CKD patients, attending a university nephrology department. Seventy-nine (79) pre-dialysis patients of disease stages 3 to 4 and 19 dialysis patients were included. HRQL was assessed by the 36-item Short-Form health survey (SF-36), defensive coping by the Rationality/Emotional Defensiveness (R/ED) scale of the Lifestyle Defense Mechanism Inventory (LDMI) and psychological distress by the depression and anxiety scales of the revised Hopkins Symptom CheckList (SCL-90-R). Regression analyses were carried out to examine the association between SF-36 dimensions and defensive coping style.

**Results:**

Patients on dialysis had worse scores on SF-36 scales measuring physical aspects of HRQL. In the fully adjusted analysis, a higher defensive coping score was significantly associated with a lower score on the mental component summary (MCS) scale of the SF-36 (worse mental health). In contrast, a higher defensive score showed a small positive association with the physical component summary (PCS) scale of the SF-36 (better health), but this was marginally significant.

**Conclusions:**

The results provided evidence that emotional defensiveness as a coping style tends to differentially affect the mental and the physical component of HRQL in CKD. Clinicians should be aware of the effects of long-term denial and could examine the possibility of screening for defensive coping and depression in recently diagnosed CKD patients with the aim to improve both physical and mental health.

## Background

Chronic kidney disease (CKD) is a life-threatening condition, persists for an extended period of time and necessitates lifelong pharmacological treatment as well as dietary restrictions. End-stage renal disease (ESRD) imposes additional constraints due to the vital need for regular sessions of renal dialysis. On these grounds, CKD and especially ESRD have been defined as provoking a state of prolonged distress [1].

Coping with the stresses of chronic disease plays a key role in determining changes in health-related quality of life (HRQL) [2]. It also reflects how well patients adjust to chronic illnesses [3]. Several authors confirmed these assumptions in spinal cord lesion [4], systemic lupus erythematosus [5] and prostate cancer [6]. These findings may have important clinical implications when considering the prognostic value of HRQL in several chronic diseases, including ESRD: decreases in HRQL have been repeatedly associated with increased morbidity and hospitalizations [7], increased mortality [7,8] as well as limited adherence to ESRD regimens [9,10] among hemodialysis (HD) patients.

Inherent in coping, emotion regulation embraces several conscious and unconscious mechanisms, involved in experiencing, perceiving, interpreting and dealing with negative emotional states [11]. Maladjustment to chronic illness has been traditionally associated with avoidance and defensive inhibition of emotions [3]. Although reduced psychological distress might be acknowledged and reported when avoidance or defensive coping is employed [12,13], evidence suggested that emotion-related physiological arousal persists [12]. Nevertheless, negative emotions remain unresolved, bringing adverse effects on both physical [12-21] and mental [14,15,22,23] health.

Early studies [24] showed evidence of defensive inhibition of emotions in HD patients, impeding psychological adjustment [25]. The greater the occurrence of the unconscious defense of denial, the more frequent the adoption of emotion-focused coping strategies among ESRD patients [1]. Systematic avoidance of disease stresses via emotion-focused coping has been associated with unfavourable mental health outcomes in peritoneal dialysis (PD) patients [26] as well as with increased mortality in HD patients [27]. These effects were independent of disease severity [26]. A recent study by Santos [28] also showed that emotion-focused coping is associated with worse physical and mental aspects of HRQL among HD patients.

Closing the mind towards threatening information may lead to silent intrapsychic tasks that adversely affect both mental [15] and physical health [12]. It is then important to evaluate the effectiveness of coping with the stresses of chronic disease. According to Roesch and Weiner [29], health related-quality of life seems a good indicator of coping effectiveness. To our knowledge, little evidence exists for coping with CKD (limited to conscious coping efforts of ESRD patients) and even less for its impact on physical and mental well-being. The present study sought to investigate the association of defensive coping with both the mental and the physical components of HRQL. Considering that not all aspects of HRQL have been found related to CKD stage [30,31] or severity [26], the present study assessed patients in different CKD stages. Psychological distress was also evaluated and controlled for together with socio-demographic and clinical variables.

## Methods

### Study design and participants

The present study was carried out at the university of Ioannina nephrology department, which offers health services to patients from north-western Greece. A cross-sectional design was employed, in order to enable comparisons among CKD patients in different disease stages. CKD stages were defined by the level of estimated glomerular filtration rate in a 24-hour urine collection (eGFR-24h), as a measure of overall kidney function [32,33].

Between March 15, 2007, and June 15, 2009, all patients attending the university of Ioannina nephrology department were consecutively included. Eligibility for the study involved all patients, who (a) were aged 20 years and older, (b) had an adequate educational level as well as an equivalent level of perception and comprehension, and (c) had read and signed the consent form. Two hundreds thirty four (234) CKD patients met the criteria. However, only 153 CKD patients agreed to participate, while 55 of them failed to appear to the scheduled appointment to fill in the questionnaires. The sample that was then formed included 98 CKD patients. Seventy-nine (79) pre-dialysis patients of disease stages 3 to 4 and 19 dialysis patients were included. None of the participants had a psychiatric diagnosis or treated with psychoactive medication before or at the time of the study.

An ethical approval for the study was obtained from the scientific committee of the university of Ioannina hospital, in accordance with the ethical standards of the Helsinki Declaration.

### Study measures

#### HRQL assessment

The 36-item Short Form health survey (SF-36) was adopted for the assessment of HRQL. It is a generic instrument, designed to measure eight health concepts, including physical functioning, bodily pain, role limitations due to physical health problems as well as due to personal or emotional problems, emotional well-being, social functioning, vitality and general health perceptions. Items from each concept are summed and rescaled with a standard range of 0 to 100, where 100 represents the best HRQL. It also provides two general indices, which refer to the physical component summary (PCS) and the mental component summary (MCS) scores. These are calculated using weights derived from a national probability sample. SF-36 has been widely used and validated by studies examining health-related perceptions, quality of life and/or depression in different medical conditions [34-36], including CKD [8,37]. SF-36 has been translated into several languages and found to possess good psychometric features [38], including the Greek version as well [39,40].

### Defensive coping assessment

Defensive coping was assessed by the Rationality/Emotional Defensiveness (R/ED) scale of the Lifestyle Defense Mechanism Inventory (LDMI). R/ED consists of 12 items, assessing individual differences in the frequency that a person engages in rational, non-emotional thought processes and behaviors (eg. "I try to understand other people even if I do not like them"; "I try to do what is sensible and logical"; etc.). Rating is based on a 4-point frequency scale, ranging from (1) "almost never" to (4) "almost always". Items are summed to give a range of 12 to 48, where 48 indicates excessive use of unconscious defensiveness (i.e., repression and denial). For its development, the Rationality/Anti-emotionality questionnaire was used, which proved to be a reliable predictor of cardiovascular disease and cancer [41,42]. Both the R/ED scale alone and the LDM inventory in total have been administered to several populations and cultures and found to possess satisfactory psychometric properties [43-46]. The Greek version of R/ED was developed, according to the recommended forward and backward translation procedure [47]. As this was the first time that the R/ED was used in a Greek patient population, reliability analysis was performed for this sample. Internal consistency was found satisfactory with a Cronbach's alpha value being 0.76.

### Psychological distress assessment

The 13-item depression scale and the 10-item anxiety scale of the revised Hopkins Symptom CheckList (SCL-90-R) were used, in order to measure psychological symptom status with a time reference of the past 7 days, including the day of assessment. The depression scale involves most typical symptoms of depressive syndromes, including emotional, cognitive and somatic correlates. The anxiety scale includes symptoms of nervousness, tension, trembling, feelings of terror and panic as well as some somatic correlates of anxiety. Items are rated on a 5-point scale of symptom distress, ranging from (0) "not all" to (4) "extremely". Items from each concept are summed and divided by the total number of responses on that concept with a range of 0 to 4, where 4 indicates extreme psychological distress. The SCL-90 inventory and its subscales have been widely used as screening tools for the initial and follow-up assessment of psychopathology in both psychiatric and medical patients [48,49]. It has shown good internal consistency and convergent validity in several clinical studies internationally [50], including Greece [51].

### Other clinical assessments and sociodemographics

Various sociodemographic and disease-related information was collected, including gender and age, educational level, employment and marital status, personal and family medical history, time of diagnosis, disease etiology and stage as well as the method and duration of dialysis treatment.

### Statistical analysis

All analyses were performed using Stata (version 9.0). Data was described as means and standard deviations (SD) for continuous variables and as frequencies and proportions for categorical variables. Pearson's *r *was applied to test for correlations among study variables. The Mann-Whitney test was used for comparisons of ranked scores. Multiple linear regression was further performed to assess the predictive power of defensive coping for explaining differences in HRQL, while adjusting for the effects of sociodemographic, clinical and psychological variables.

## Results

From the 234 patients eligible for inclusion, 136 refused (81 immediately, while 55 patients although initially agreed did not appear to the scheduled appointment). Patients who refused did not differ in gender compared to patients included in the study (43% *vs *41% female patients, p = 0.54), but they tended to be older (mean age 57 *vs *54, p = 0.04). They were also more likely to be in pre-dialysis stages. For those who refused, 88% were in the pre-dialysis stage (120/136) and 12% were in ESRD (16/136), while for those who took part, the figures were 81% (79/98) and 19% (19/98) respectively (p = 0.03).

Table 1 presents the sociodemographic, clinical and psychological characteristics of the included sample. Most patients were married (86.7%) males (59%), being at a pre-dialysis stage (80.6%). Their ages ranged from 20 to 80 years (53.7 ± 11.5). Less than half of the patients were currently employed (42.9%). Their education ranged from 6 to 16 years (10 ± 3.7). The diagnosis of CKD had been established more than three years before the conduction of the study for 78.5% of patients and the underlying cause was identified as non-diabetic nephropathy for 76.5% of patients. The mean score for defensive coping was 37.07(±5.17), for depression 0.96(±0.56) and for anxiety 0.48(±0.46).

**Table 1:** Sociodemographic, clinical and psychological characteristics in chronic kidney disease patients (N = 98).

SOCIODEMOGRAPHIC, CLINICAL AND PSYCHOLOGICAL VARIABLES		N (%) or mean (SD)
**GENDER**	Male	**58**(59.18*%*)
	Female	**40**(40.82%)
		
**AGE**	20-39 yrs	**6**(6.12%)
	40-59yrs	**53**(54.08%)
	>60 yrs	**39**(39.80%)
		
**EDUCATION**	6 yrs	**37**(37.76%)
	9 yrs	**15**(15.30%)
	12 yrs	**26**(26.54%)
	>12 yrs	**20**(20.40%)
		
**MARITAL STATUS**	Married	**85**(86.74%)
	Single	**6**(6.12%)
	Divorced	**6**(6.12%)
	Widower	**1**(1.02%)
		
**EMPLOYMENT STATUS**	Employed	**42**(42.86%)
	Unemployed	**2**(2.04%)
	Retired	**31**(31.63%)
	Domestic	**21**(21.43%)
	Student	**2**(2.04%)
		
**CLINICAL VARIABLES**	**Disease stage**	
	Pre-dialysis	**79**(80.61%)
	Dialysis	**19**(19.39%)
	**Time since diagnosis**	
	<1 yr	**1**(1.02%)
	1-3 yrs	**20(**20.41%)
	>3 yrs	**77**(78.57%)
	**CKD etiology**	
	Diabetic nephropathy	**23**(23.47%)
	Non-diabetic nephropathy	**75**(76.53%)
		
**PSYCHOLOGICAL VARIABLES**	**Defensive coping score**^a^	**37.07**(5.17)
	**Depression score**^b^	**0.96**(0.56)
	**Anxiety score**^c^	**0.48**(0.46)

^a ^Scores on the Rationality/Emotional Defensiveness (R/ED) scale (range: 12-48) of the Lifestyle Defense Mechanism Inventory (LDMI).

^b,c ^Scores on the depression (range: 0-4) and the anxiety (range: 0-4) scales of the 90-item revised Hopkins Symptom CheckList (SCL-90-R).

Table 2 shows the mean scores and SDs on the SF-36 dimensions by the time since the diagnosis of CKD and by disease stage. Patients who were diagnosed with CKD more than 3 years ago and dialysis patients had significantly lower scores on PCS (*p *< 0.001), including physical functioning (*p *< 0.05 and *p *< 0.001 respectively), bodily pain (*p *< 0.001 and *p *< 0.05 respectively) and general health (*p *< 0.001 and *p *< 0.01 respectively). Nonsignificant differences were obtained for MCS. Only patients who were diagnosed more than 3 years ago reported a significant greater impairment of social functioning (*p *< 0.01).

**Table 2:** Mean SF-36 scores by time since diagnosis and disease stage in chronic kidney disease patients (N = 98).

	TIME SINCE DIAGNOSIS Mean(SD)		DISEASE STAGE Mean(SD)		
SF-36 DIMENSIONS^a^					
	≤3 years (N = 21)	>3 years (N = 77)	Pre-dialysis (N = 79)	Dialysis (N = 19)	Total (N = 98)
PCS^b^	**51.97(7.06)**	45.24(8.18) ^***^	**48.32(7.40)**	39.87(8.99) ^***^	46.68(8.39)
MCS^c^	45.20(10.15)	47.75(10.54)	47.52(10.25)	45.90(11.48)	47.20(10.46)
Physical functioning	**82.61(18.61)**	74.67(18.99) ^*^	**80.44(15.19)**	59.47(24.31) ^***^	76.37(19.10)
Role/Physical	59.52(42.18)	74.35(36.49)	73.42(35.65)	61.84(46.67)	71.17(38.05)
Bodily pain	**94.33(13.53)**	71(28.22) ^***^	**79.59(23.63)**	61.05(36.76) ^*^	76.00(27.46)
General health	**67.95(19.73)**	50.53(17.92) ^***^	**57.48(18.92)**	40.89(16.77) ^**^	54.26(19.58)
Vitality	63.33(23.62)	59.87(20.35)	61.77(20.20)	55.78(24.10)	60.61(21.02)
Social functioning	**86.90(16.52)**	72.07(24.90) ^**^	77.84(22.37)	64.47(28.34)	75.25(24.07)
Role/Emotional	58.73(37.86)	74.02(36.52)	74.68(34.26)	54.38(44.73)	70.74(37.15)
Mental Health	64.76(18.57)	69.76(20.31)	68.75(19.73)	68.42(21.49)	68.69(19.97)

^a ^Lower scores indicate worse health; ^b^PCS: Physical Component Summary score; ^c^MCS: Mental Component Summary score

*p <.05, **p <.01 and ***p <.001, Mann-Whitney U test for comparisons of ranked scores.

The correlation coefficients for SF-36 dimensions, defensive coping, depression and anxiety are presented on Table 3. Significant negative associations were obtained for PCS, depression and anxiety (*p *< 0.01) as well as for MCS, defensive coping, depression and anxiety (*p *< 0.01). It is noted that defensive coping was not associated with PCS but it was significantly associated with depression (*p *< 0.05).

**Table 3:** Pearson's correlation coefficients among SF-36 physical component summary (PCS) and mental component summary (MCS), defensive coping score, depression score and anxiety score in chronic kidney disease patients (N = 98).

	1	2	3	4	5
**1. Physical component summary**	1				
**2. Mental component summary**	0.284**	1			
**3. Defensive coping score**^a^	0.039	-0.384**	1		
**4. Depression score**^b^	-0.532**	-0.738**	0.236*	1	
**5. Anxiety score**^c^	-0.394**	-0.554**	0.065	0.638**	1

^a ^Scores on the Rationality/Emotional Defensiveness (R/ED) scale (range: 12-48) of the Lifestyle Defense Mechanism Inventory (LDMI).

^b,c ^Scores on the depression (range: 0-4) and the anxiety (range: 0-4) scales of the 90-item revised Hopkins Symptom CheckList (SCL-90-R).

*p < 0.05, **p < 0.01

### Association between SF-36 dimensions and defensive coping scores

Results of the regression analysis are shown in Table 4. In the fully adjusted model, a higher defensive coping score was significantly associated with a lower MCS score (worse mental health) but a higher PCS score (better physical health), although the latter was marginally significant (*b *= 0.26, *p *= 0.046). Since in the univariate analysis (Table 3) there was no association between defensive coping and PCS scores, we repeated the analysis by excluding depression to assess the possibility that the latter had a negative confounding effect on the association between PCS and defensive coping. Indeed, the analysis showed that this was true, since the beta coefficient for defensiveness was reduced in this analysis (*b *= 0.19, *p *= 0.23). *R*^*2*^- values for the fully adjusted PCS and MCS models were 0.70 and 0.73 respectively.

**Table 4:** Association of SF-36 physical component summary (PCS) and mental component summary (MCS) scores with sociodemographic, clinical and psychological variables.

DEPENDENT VARIABLES	INDEPENDENT VARIABLES	***B *(95% CI)**^a^
**PCS**^b^	**Depression score**^c^	-7.96 (-10.65, -5.26)***
	**Anxiety score**^d^	-0.54 (-2.73, 3.82)
	**Defensive coping score**^e^	0.26 (0.004, 0.51)*
	**Age**	-.07 (-0.25, 0.10)
	**Female**^f^	- 6.38 (-9. 64, -3.13)***
	**Dialysis**^g^	- 8.48 (-11.28, -5.68)***
	**>3 years since diagnosis**^h^	-1.94 (-5.49, 1.59)

**MCS**^b^	**Depression score**^c^	-9.15 (-12.31, -5.99)***
	**Anxiety score**^d^	-6.35 (-10.19, -2.51)**
	**Defensive coping score**^e^	-0.51 (-0.81, -0.21)**
	**Age**	0.13 (-0.06, 0.34)
	**Female**^f^	8.55 (4.74, 12.36)***
	**Dialysis**^g^	-3.37 (-6.65, -0.09)*
	**>3 years since diagnosis**^h^	6.25 (2.09, 10.40) **

^a ^Beta coefficients (95% Confidence Intervals) from linear regression models adjusted for education, marital status, employment status and all variables listed on table.

^b ^Lower scores indicate worse health

^c,d^ Scores on the depression (range: 0-4) and the anxiety (range: 0-4) scales of the 90-item revised Hopkins Symptom CheckList (SCL-90-R).  e Scores on the Rationality/Emotional Defensiveness (R/ED) scale (range: 12-48) of the Lifestyle Defense Mechanism Inventory (LDMI)

^e ^Scores on the Rationality/Emotional Defensiveness (R/ED) scale (range: 12-48) of the Lifestyle Defense Mechanism Inventory (LDMI).

^f ^*Vs *male

^g ^*Vs *pre-dialysis patients

^h ^*Vs *≤3 years since diagnosis

^* ^*p *< 0.05, ^** ^*p *< 0.01, ^*** ^*p *< 0.001

## Discussion

HRQL has been considered as a good indicator of physical and psychological well-being in chronic disease [29] as well as a strong predictor of adherence [9,10], morbidity [7] and mortality [7,8] in ESRD. It has been argued that perceived impairment of HRQL is mediated by patients' efforts to cope with the stresses of the disease [26]. Maintaining a satisfactory level of quality of life is then associated with effective coping [29].

Defensive coping, as introduced by S. Freud [52], represents a distortion of unwelcome reality [53]: it alters the way a stressful situation is perceived, by expelling disturbing thoughts or emotions from conscious awareness. Its functions resemble the ones of medical defense mechanisms but in regard to mental health [13]. Physical disease is considered to evoke defense-related emotions, such as fear, anxiety, sadness and anger [13]. Thus, the role of psychological defense is to guard individuals against disease-related information that induces distress and threatens psychological equilibrium. In the context of physical health conditions, evidence suggested that extended use of emotional defensiveness (e.g. denial) might be palliative but often at the cost of mental health [14,15,22,23] and, in the long run, of physical health [12-21] as well.

The present study aimed to investigate the association of emotional defensiveness as a coping style with the mental and physical components of HRQL among CKD patients in different stages. After controlling for psychological distress, sociodemographic and disease-related variables, our findings revealed that emotional defensiveness was indeed an explanatory variable for HRQL. However it had a distinct, contrasting impact on mental and physical outcomes.

With regard to mental well-being, the present study provided evidence that greater use of emotional defensiveness is associated with more depressive symptoms and a deteriorated mental component of HRQL. These adverse effects of defensive coping were apparent, regardless of disease stage and the time that occurred since CKD was diagnosed. Previous studies also showed that both conscious and unconscious avoidance of disease stresses is associated with increased depressive symptoms and a poor total mental health functioning in various physical disorders [22,23], including ESRD [25,26,28]. A high prevalence of depressive symptoms has been also systematically recorded in CKD patients [54]. These homogeneous findings are theoretically meaningful in that, defending against painful emotions impedes the process of grief and gives rise to essential depression. While grief involves working on disease-related loss (e.g. loss of independency, self-image, etc.) and reaching an adaptive resolution [55], depression reflects the tendency to defensively avoid (e.g. denial) loss-related emotions or information; it represents an absence of desire and intrapsychic life [56], leading to a rather maladaptive resolution [55].

However, these adverse effects of defensive coping were restricted to mental well-being only. It was interesting to find that emotional defensiveness tended to predict a slightly better physical aspect of HRQL. In the unadjusted association though (see table 3), emotional defensiveness was not associated with PCS, a finding which is explained by the negative confounding effect of depression on this association. It should be noted, however, that the adjusted association of emotional defensiveness and PCS was marginally significant. Therefore, the most robust finding of the present study was the absence of a negative effect of defensive coping on physical well-being.

Opposed to this, most findings so far suggested that patients with several health conditions, using long-term avoidant or defensive coping, tended to report a deteriorated physical well-being [19,57]. When the present research team examined the same psychological variables as here in patients with essential hypertension, a trend for a negative association was observed for emotional defensiveness and PCS. It could be assumed then that the present finding might be disease specific: for CKD patients, defensive coping may influence mental and physical well-being in conflicting ways. Taylor and her colleagues [58] arrived at similar conclusions when examined patients awaiting lung transplantation: active coping was associated with better physical health in chronic obstructive pulmonary disease but not in cystic fibrosis.

CKD is a health condition that persists over an extended period of time and brings no obvious symptoms or signs almost till the end stage. One could assume then that closing the mind towards the potential threats of the disease is further facilitated by this silent progress of CKD. Such an assumption may gain grounds, when considering that the study sample consisted mainly of pre-dialysis patients, who reported a significantly higher physical aspect of HRQL compared to dialysis patients. Therefore, perceived physical health appears optimal, since limitations due to health condition are easy to ignore in pre-dialysis stages, although at the cost of mental well-being. For ESRD though, previous research did establish a relationship between emotion-focused coping and a deteriorated physical aspect of HRQL [28]. The adoption of emotion-focused strategies has been also positively associated with defensive coping (i.e. denial) among ESRD patients [1]. On these grounds, it might be appropriate to consider our finding in relation with CKD stage: the effects of emotional defensiveness on perceived physical health may not apply to ESRD, due to the small number of the dialysis patients enrolled. Future research is certainly needed to clarify this association with regard to CKD stage.

While no negative effects of emotional defensiveness were observed on perceived physical health at least for pre-dialysis patients, it has not been possible to conclude whether this finding also applies to actual physical health, due to the lack of relevant data for the study sample. Previous findings suggested that, in the long run, defensive coping may adversely affect actual physical health through certain patterns of behaviour. Devaluation of physical symptoms, failure to seek and comply with medical recommendations and rehabilitation as well as an increased risk of comorbidity and mortality have all been associated with defensive coping in several chronic disease [18-21]. Limited adherence to ESRD management has been repeatedly observed in dialysis patients as well [59]. These patterns of behaviour might be indeed indicative of the maladaptive functions of defensive denial [14]. Disease-related information (i.e. physical symptoms and medical recommendations) might have been subject to defensive functioning and thus overlooked. On these grounds, it seems worthwhile for future research to examine associations of emotional defensiveness and actual physical health in both cross-sectional and prospective studies.

In summary, the results of the present study indicated that emotional defensiveness is differentially associated with mental and physical components of HRQL in CKD: perceived mental health tended to deteriorate, while perceived physical health tended to slightly improve. These findings may have important implications for the management of CKD patients as well as the orientation of psychosocial interventions. It might be important for clinicians to consider that pre-dialysis patients with good physical health do not necessarily have good mental health as well. In addition, physical health may also deteriorate as effects of long-term emotional defensiveness become apparent. Succeeding a moderate defensive coping with CKD may indeed facilitate psychological adjustment and possibly promote physical health as well. Future research is certainly needed to confirm and further examine this disease-specific impact of defensive coping on health related quality of life.

## Limitations

The findings of the present study should be considered in the context of certain limitations. First, this was a cross-sectional study and, therefore, issues of temporal association cannot be examined. Although it is anticipated that coping style is a relatively stable personality characteristic, we do not have data on the stability of the defensive coping style and we cannot exclude the possibility that the severity of the disease may influence the coping style or modify its characteristics. Second, no objective data regarding CKD severity and/or comorbid conditions was collected for the study sample and, thus, it is not possible to examine associations of emotional defensiveness and actual physical health condition. Third, the sample may have not efficiently represented CKD and especially ESRD patients in Greece, as a result of including a small number of patients from a single academic centre. Hence, the generalisability of the results might be somewhat limited. In addition, it is not possible to conclude whether the effects of emotional defensiveness on perceived physical health also apply to ESRD and to generalize our findings to the broader population of dialysis patients in Greece. Another factor that may have limited the external validity of the results is the proportion of CKD patients accepting to take part. We found no differences in gender between those who refused and those who accepted to take part. However, it was more likely for patients who refused to be slightly older as well as in pre-dialysis stages. There is no indication though that the reason for their refusal was related to the study aims, in order to introduce selection bias to the study. Participation to the study was voluntary and most of the patients that refused reported lack of time or interest. To our opinion, patients who refused appeared less motivated to take part due to the fact that additional research was carried out at that time and they were asked to participate to all.

## Conclusions

The present study investigated the association between emotional defensiveness as a coping style and perceived impairment of physical and mental well-being in CKD. Psychological distress, sociodemographic and clinical variables were adjusted to isolate the impact of emotional defensiveness. The results confirmed previous findings, suggesting that defensive coping does relate to worse mental components of HRQL. However, no negative effects of emotional defensiveness were observed for the physical aspects of HRQL. Instead, a marginally significant positive effect was obtained. Although these findings were liable to certain limitations, they have still posed several issues to consider in both clinical practice and future research: good physical health may not be necessarily accompanied by good mental health and physical health may worsen as a result of long-term emotional defensiveness. Assessment of defensive coping and depression in recently diagnosed CKD patients may be warranted to improve both physical and mental health of CKD patients.

## Abbreviations

CKD: Chronic Kidney Disease; HRQL: Health-Related Quality of Life; ESRD: End Stage Renal Disease; PD: Peritoneal Dialysis; HD: Haemodialysis; eGFR-24h: Estimated Glomerular Filtration Rate - 24 hour urine collection; SF-36: the 36-item Short Form health survey; PCS: Physical Component Summary; MCS: Mental Component Summary; SCL-90-R: the 90-item Revised Hopkins Symptom CheckList; LDMI: the Lifestyle Defense Mechanism Inventory; R/ED: Rationality/Emotional Defensiveness; CI: Confidence Intervals.

## Competing interests

The authors declare that they have no competing interests.

## Authors' contributions

AK collected and interpreted the data, drafted and included revisions to the manuscript. PS conceived the research question, performed the statistical analyses, interpreted the data and critically reviewed the manuscript. DD conceived of, designed and coordinated the study and critically reviewed the manuscript. MI and RK assisted with the collection of the data. VM commented on the final draft. KCS designed and coordinated the study and critically reviewed the manuscript. All authors have read and approved the final manuscript.

## Pre-publication history

The pre-publication history for this paper can be accessed here:

http://www.biomedcentral.com/1471-2369/12/28/prepub
